# Association of cigarette smoking with increased use of heated tobacco products in middle-aged and older adults with self-reported chronic obstructive pulmonary disease, asthma, and asthma-COPD overlap in Japan, 2022: the JASTIS study

**DOI:** 10.1186/s12890-023-02668-1

**Published:** 2023-09-30

**Authors:** Shingo Noguchi, Tomohiro Ishimaru, Yoshihisa Fujino, Kazuhiro Yatera, Takahiro Tabuchi

**Affiliations:** 1 Department of Respiratory Medicine, Tobata General Hospital, 1-3-33, Fukuryugi, Tobata-Ku, Kitakyushu-City, Fukuoka, 804-0025 Japan; 2 Department of Respiratory Medicine, University of Occupational and Environmental Health, Japan, 1-1 Iseigaoka, Yahatanishi-Ku, Kitakyushu-City, Fukuoka, 807-8555 Japan; 3 Department of Environmental Epidemiology, Institute of Industrial Ecological Sciences, University of Occupational and Environmental Health, Japan, 1-1 Iseigaoka, Yahatanishi-Ku, Kitakyushu-City, Fukuoka, 807-8555 Japan; 4 Cancer Control Center, Osaka International Cancer Institute, 3-1-69 Otemae, Chuo-Ku, Osaka, 541-8567 Japan

**Keywords:** Asthma-COPD overlap, Chronic obstructive pulmonary disease, Dual use, Heated tobacco products, Smoking cessation

## Abstract

**Background:**

Smoking cessation is the most important intervention in chronic obstructive pulmonary disease (COPD), asthma, and asthma-COPD overlap (ACO); however, high rates of current cigarette smoking are observed in adults with these respiratory diseases. Meanwhile, rapidly increasing use of heated tobacco products (HTPs) is observed in Japan; however, the status of HTPs use has not been fully understood in adults with COPD, asthma, and ACO. This study aimed to reveal the association between COPD, asthma, and ACO and HTPs use in adults.

**Methods:**

Data on Japanese individuals ≥ 40 years old obtained from the Japan Society and New Tobacco Internet Survey were analyzed. The prevalence of HTPs use in adults with COPD, asthma, and ACO, among individuals categorized into three groups according to cigarette smoking (never, former, and current), was calculated and the relationship between each disease and HTPs use were evaluated. The clinical diagnosis of these diseases was based on the self-reported diagnosis, as obtained from questionnaires.

**Results:**

A total of 19,308 individuals were included. The proportions of never, past, and current cigarettes smokers were 10,900 (56.5%), 4,903 (25.4%), and 3,505 (18.2%), respectively, and that of HTPs use was 1,813 (9.4%). In current cigarettes smokers, the adjusted odds ratios (ORs) of HTPs use was 2.88 (95% CI [confidence interval], 1.86–4.47), 1.23 (95% CI, 0.99–1.52), and 5.81 (95% CI, 3.12–10.82) in adults with COPD, asthma, and ACO compared to those without these respiratory diseases, respectively. Meanwhile, in past cigarettes smokers, the adjusted ORs of HTPs use was 0.51 (95% CI, 0.24–1.08), 0.69 (95% CI, 0.53–0.88), and 0.25 (95% CI, 0.06–1.07) in adults with COPD, asthma, and ACO, respectively.

**Conclusions:**

HTPs use is more prevalent among current cigarettes smokers with COPD, asthma, and ACO compared to those without these respiratory diseases. Complete cessation of smoking both cigarettes and HTPs is the only way to achieve complete smoking cessation, therefore, adults with COPD, asthma, and ACO need to make greater efforts to quit smoking.

## Introduction

Cigarette smoking is widely known to be harmful for adults with chronic obstructive pulmonary disease (COPD) and asthma. Cigarette smoking is the most important risk factor in the pathogenesis and progression of COPD and causes a decline of forced expiratory volume in one second (FEV_1.0_) and an increase in COPD exacerbation and/or mortality [1–4]. Additionally, it leads to poor symptom control, decreased FEV_1.0_, an attenuating effect of corticosteroid therapy, and increase in acute exacerbation in adults with asthma [5, 6]. Despite the above effects, high rates of current cigarette smoking are observed in adults with COPD (12.5%–45.1%) and asthma (5.8%–25.7%) [7–10].

Heated tobacco products (HTPs) are electronic devices that heat tobacco leaves, and aerosols from HTPs include nicotine, carcinogens, additives, and flavored substances [11]. Japan is one of the first countries to launch HTPs and is also one of the major countries for HTPs sales worldwide [12, 13]. Short and long-term adverse effects of HTPs use remain unelucidated [14]; however, some studies reported the adverse effects of HTPs related to endothelial function, oxidative stress, and platelet activation, similar to those observed with traditional cigarette smoking [15, 16]. Additionally, HTPs are associated with the pathogenesis of pulmonary emphysema in mice [17] and poor respiratory symptoms in clinical practice [18, 19].

According to the Japan “Society and New Tobacco” Internet Survey (JASTIS) study, the number of HTP users in Japan increased about 50-fold from 0.2% in 2015 to 10.9% in 2020 [20, 21]. The Japanese cigarette makers promote HTPs to quit smoking in spite of claim that their use itself leads to a strong association for relapse of and/or restarting regular cigarette smoking [13]. Smoking cessation is the most important intervention for adults with chronic respiratory diseases, such as COPD and asthma [10, 22]; however, the status of HTPs use in adults with these respiratory diseases remains unelucidated. In addition, the frequencies of HTPs use also vary by age group and the prevalence of HTPs use is significantly different between adults 40 years or older and those under 40 years old in Japan [21, 23]. To the best of our knowledge, no reports targeting the association between the status of HTPs use and COPD and asthma in middle-aged and older adults exist.

In the present study, we enrolled adults over 40 years old and aimed at revealing the current status of HTPs use in adults with COPD, asthma, and asthma and COPD overlap (ACO), which is newly proposed and characterized by persistent airflow limitation with overlapping features of COPD and asthma [24, 25], using internet survey.

## Methods

### Data source

The present study aimed to reveal the current status of HTPs use in adults aged over 40 years with COPD, asthma, and ACO. It was conducted with the data from the JASTIS, an ongoing longitudinal internet cohort study designed to investigate the prevalence and different ways of smoking conventional cigarettes, HTPs, and e-cigarettes in Japan and was also recorded information on the demographics, socioeconomic status, and health conditions of participants [26]. Briefly, participants were randomly recruited from the total number of major nationwide large survey panels (Rakuten Insight, Inc., Tokyo, Japan) with a pool of 2.3 million panelists covering all social categories as defined by the Japanese census, and the survey was continued until the number of participants who completed the questionnaire reached the target number. Participants answered the questionnaire after reading the survey explanation and provided web-based written informed consent on registration. The JASTIS 2022 survey was performed between February 1, 2022, to February 28, 2022. Using its data, the study was approved by the Research Ethics Committee of the Osaka International Cancer Institute (No. 1412175183) and the National Institute of Public Health (NIPH-IBRA#12112), and was performed according to the Declaration of Helsinki.

### Inclusion and exclusion criteria

The data of all responders (33,000) of the 2022 survey, except those whose responses were inconsistent with the information in the earlier survey (2015 to 2021), were included. Briefly, 28,776 participants were recruited from the follow-up survey (response rate 71.9%), and 4,224 participants with aged 18–49 were newly collected from the panel in the 2022 survey. Further detailed information is described in the study profile [26]. In this study, responders under 40 years old and those with straight-lining or discrepancies were excluded from this study to maintain the validity of the survey. Invalid responses were defined as follows: not choosing the second answer from the bottom after the question “Please choose the second option from the bottom out of five options”; reporting use of all drug in the question “Do you use either drug out of eight items”; and having all items in the question “Do you currently have any chronic diseases”, similar to a previous report [26].

### Definition of COPD, asthma, and ACO

Participants were asked the question, “Do you currently have any chronic diseases?” and were defined to have COPD or asthma when they confirmed of having either one of them, and to have ACO when they confirmed on having both COPD and asthma.

### Definition of cigarettes and HTPs use

The types of tobacco products were classified as follows: cigarette use (paper-wrapped and/or roll-your-own cigarettes) and HTPs (Ploom TECH, IQOS, glo, and/or lil HYBRID). Participants were asked two questions, “Have you used each tobacco product during the past 30 days?” and “How many cigarettes do (did) you use per day at present (or in the past)?”, and they were categorized into three groups according to the following definitions: individuals who had used cigarettes in the past 30 days were classified as “current cigarettes smokers”; individuals who had ever smoked more than one cigarette and who had not used cigarettes in the past 30 days were classified as “former cigarettes smokers”; and individuals who had never smoked a cigarette before were classified as “never cigarettes smokers”*.* The participants were also asked the same two questions about HTPs use and those who had used HTPs in the past 30 days selected “HTPs use”, all other respondents selected “no HTPs use”, as described in a previous report [11].

### Other variables

Participants were classified into four age groups (40–49, 50–59, 60–69, and 70–81 years). Educational attainment was classified into three groups; junior high or high school, vocational school or college, and university or graduate school. Equivalent household income was calculated by the household income divided by the square root of the household, and the population was divided into quartiles, and the 25th, median, and 75th equivalent household income of the study population were 1,500, 3,000, and 4,500 thousand-yen, respectively. The drinking status was divided into three groups; never, occasionally, and regular, as a previous report [11].

### Statistical analyses

First, respondents were stratified into three groups previously defined, and the number and percent of demographic characteristics were calculated for each. Second, in each stratum, we evaluated the association between the current history of COPD, asthma, or ACO and HTPs use with the Chi-square test or Fisher’s exact test. Finally, multivariable logistic regression analyses were performed to calculate adjusted odds ratios (ORs) and 95% confidence intervals (CIs) for these associations. We adjusted for sex, age, educational attainment, equivalent household income, and alcohol intake according to the relevant study [11]. All *P*-values were two-sided, and the statistical significance was set at *P *< 0.05. We used Stata/SE 16.1 (Stata, Corp, College Station, TX, USA) for all analyses.

## Results

**Fig. 1:**
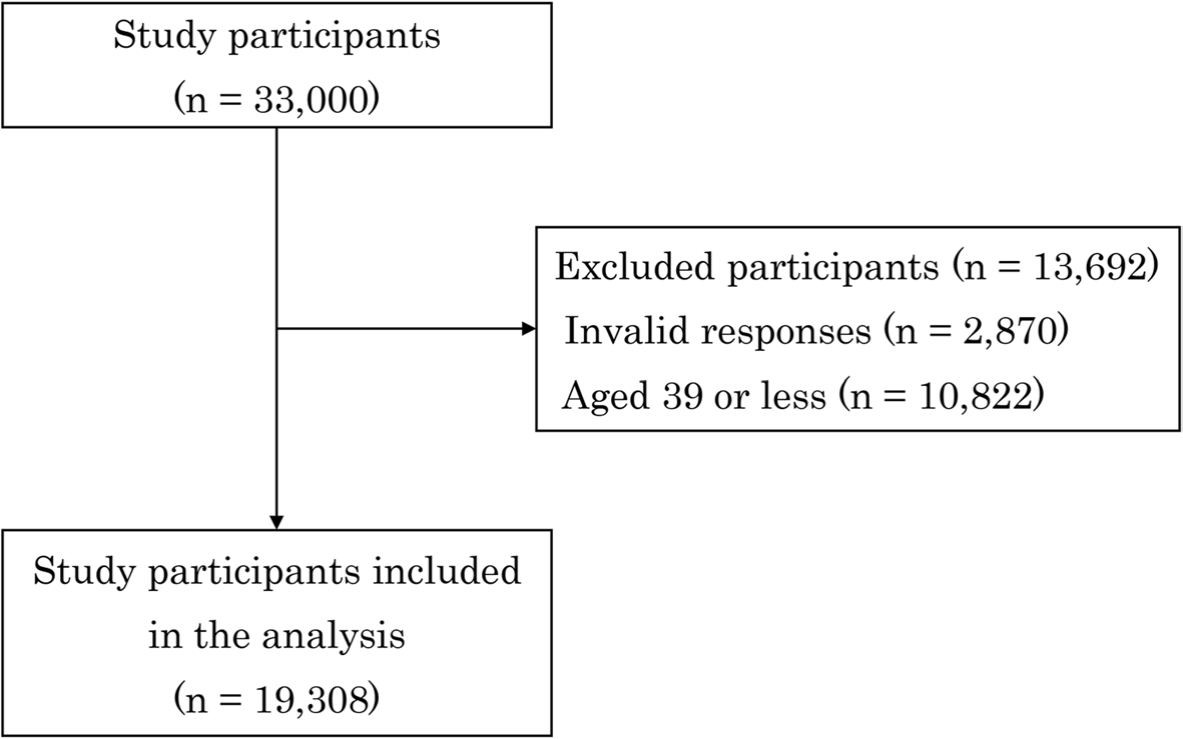
This figure illustrates the entry flow chart

Among 33,000 participants, 10,822 of ≤ 39 years of age and 2,870 with invalid responses were excluded and a total of 19,308 adults of ≥ 40 years of age were eventually included in this study (Fig. 1). The demographic characteristics of these adults are summarized in Table 1. Among these 19,308 adults, 9,679 (50.1%) were women, and 9,629 (49.9%) were men. The proportions of never, past, and current cigarettes smokers were 10,900 (56.5%), 4,903 (25.4%), and 3,505 (18.2%), respectively. Meanwhile, the number of adults who used HTPs was 1,813 (9.4%) as a whole, and the usage for each type of HTPs was as follows: 809 (4.2%) for Ploom TECH, 919 (4.8%) for IQOS, 712 (3.7%) for glo, and 110 (0.6%) for lil HYBRID. The incidence of COPD, asthma, and ACO were 258 (1.3%), 2,327 (12.1%), and 113 (0.6%), respectively.

**Table 1:** Demographic characteristics of participants

	Never cigarettes smokers				Past cigarettes smokers				Current cigarettes smokers				Total	
	HTPs (-)		HTPs ( +)		HTPs (-)		HTPs ( +)		HTPs (-)		HTPs ( +)			
	(*n* = 10,809)		(*n* = 91)		(*n* = 4,140)		(*n* = 763)		(*n* = 2,546)		(*n* = 959)		(*n* = 19,308)	
Sex														
Women	7,328	(67.8)	31	(34.1)	1,104	(26.7)	192	(25.2)	814	(32.0)	210	(21.9)	9,679	(50.1)
Men	3,481	(32.2)	60	(65.9)	3,036	(73.3)	571	(74.8)	1,732	(68.0)	749	(78.1)	9,629	(49.9)
Age (years)														
40–49	3,028	(28.0)	37	(40.7)	829	(20.0)	313	(41.0)	727	(28.6)	390	(40.7)	5,324	(27.6)
50–59	2,440	(22.6)	28	(30.8)	892	(21.5)	244	(32.0)	713	(28.0)	300	(31.3)	4,617	(23.9)
60–69	2,552	(23.6)	21	(23.1)	1,180	(28.5)	157	(20.6)	634	(24.9)	205	(21.4)	4,749	(24.6)
70–81	2,789	(25.8)	5	(5.5)	1,239	(29.9)	49	(6.4)	472	(18.5)	64	(6.7)	4,618	(23.9)
Educational attainment														
Junior high or high school	3,409	(31.5)	23	(25.3)	1,273	(30.7)	258	(33.8)	864	(33.9)	297	(31.0)	6,124	(31.7)
Vocational school or college	2,938	(27.2)	23	(25.3)	761	(18.4)	156	(20.4)	519	(20.4)	159	(16.6)	4,556	(23.6)
University or graduate school	4,462	(41.3)	45	(49.5)	2,106	(50.9)	349	(45.7)	1,163	(45.7)	503	(52.5)	8,628	(44.7)
Equivalent household income (JPY)														
< 1,500,000	1,777	(16.4)	6	(6.6)	752	(18.2)	76	(10.0)	377	(14.8)	115	(12.0)	3,103	(16.1)
1,500,000–2,999,999	2,717	(25.1)	28	(30.8)	1,137	(27.5)	163	(21.4)	626	(24.6)	184	(19.2)	4,855	(25.1)
3,000,000–4,499,999	1,769	(16.4)	19	(20.9)	741	(17.9)	175	(22.9)	495	(19.4)	233	(24.3)	3,432	(17.8)
≥ 4,500,000	2,023	(18.7)	25	(27.5)	784	(18.9)	212	(27.8)	545	(21.4)	304	(31.7)	3,893	(20.2)
Unknown	2,523	(23.3)	13	(14.3)	726	(17.5)	137	(18.0)	503	(19.8)	123	(12.8)	4,025	(20.8)
Alcohol intake														
Never	5,737	(53.1)	39	(42.9)	1,304	(31.5)	270	(35.4)	950	(37.3)	315	(32.8)	8,615	(44.6)
Occasionally	3,430	(31.7)	31	(34.1)	1,205	(29.1)	219	(28.7)	662	(26.0)	299	(31.2)	5,846	(30.3)
Regular	1,642	(15.2)	21	(23.1)	1,631	(39.4)	274	(35.9)	934	(36.7)	345	(36.0)	4,847	(25.1)
Type of HTP														
Ploom TECH	-		31	(34.1)	-		236	(30.9)	-		542	(56.5)	809	(4.2)
IQOS	-		42	(46.2)	-		391	(51.2)	-		486	(50.7)	919	(4.8)
glo	-		26	(28.6)	-		255	(33.4)	-		431	(44.9)	712	(3.7)
lil HYBRID	-		4	(4.4)	-		4	(0.5)	-		102	(10.6)	110	(0.6)
COPD	64	(0.6)	2	(2.2)	95	(2.3)	8	(1.0)	44	(1.7)	45	(4.7)	258	(1.3)
Asthma	1,193	(11.0)	7	(7.7)	547	(13.2)	83	(10.9)	336	(13.2)	161	(16.8)	2,327	(12.1)
ACO	18	(0.2)	0	(0.0)	43	(1.0)	2	(0.3)	16	(0.6)	34	(3.5)	113	(0.6)

*ACO* Asthma and COPD Overlap, *COPD* Chronic obstructive pulmonary disease, *HTPs* Heated tobacco products, *JPY* Japanese Yen

**Fig. 2:**
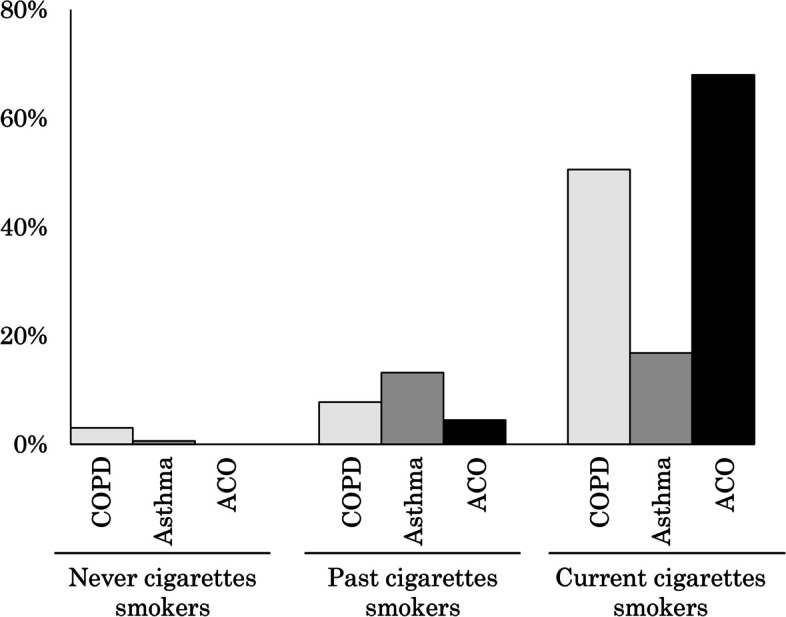
HTPs use in COPD, asthma, and ACO adults by never, past and current smoking status

The proportion of HTPs use in adults with COPD, asthma, and ACO as per the cigarette smoking status is shown in Fig. 2. The proportions of HTPs use were 2 (3.0%), 7 (0.6%), and 0 (0.0%) in never cigarettes smokers, 8 (7.8%), 83 (13.2%), and 2 (4.4%) in past cigarettes smokers, and 45 (50.6%), 161 (16.8%), and 34 (68.0%) in those with COPD, asthma, and ACO, respectively.

The results of the analyzed associations between HTPs use and COPD, asthma, and ACO by each cigarette smoking status are demonstrated in Table 2. After adjusting for sex, age, educational attainment, equivalent household income, and alcohol intake, the adjusted ORs and 95% CIs of HTPs use in never cigarettes smokers were 3.64 (0.86–15.46) for COPD and 0.61 (0.28–1.33) for asthma compare to those without each disease, respectively. No significant differences were reported between each disease and HTPs use.

**Table 2:** Association between COPD, Asthma, and ACO and HTPs use

	Univariate			Adjusted^a^		
	OR	(95% CI)	*P*-value	OR	(95% CI)	*P*-value
Never cigarettes smokers						
COPD	3.77	(0.91–15.65)	0.105*	3.64	(0.86–15.46)	0.08
Asthma	0.67	(0.31–1.46)	0.31	0.61	(0.28–1.33)	0.215
ACO	-	-	-	-	-	-
Past cigarettes smokers						
COPD	0.45	(0.22–0.93)	0.027	0.51	(0.24–1.08)	0.078
Asthma	0.80	(0.63–1.02)	0.077	0.69	(0.53–0.88)	0.003
ACO	0.25	(0.61–1.04)	0.021*	0.25	(0.06–1.07)	0.062
Current cigarettes smokers						
COPD	2.80	(1.84–4.27)	< 0.001	2.88	(1.86–4.47)	< 0.001
Asthma	1.33	(1.08–1.63)	0.007	1.23	(0.99–1.52)	0.057
ACO	5.81	(3.19–10.58)	< 0.001	5.81	(3.12–10.82)	< 0.001

*ACO* Asthma and COPD Overlap, *COPD* Chronic obstructive pulmonary disease, *HTPs* Heated tobacco products, *OR* odds ratio, *CI* confidence interval

^*^Fisher’s exact test

^a^Adjusted for sex, age, educational attainment, equivalent household income, and alcohol intake

After adjusting for confounders, the adjusted ORs and 95% CIs of HTPs use in past cigarettes smokers were 0.51 (0.24–1.08) for COPD, 0.69 (0.53–0.88) for asthma, and 0.25 (0.06–1.07) for ACO compared to those without each disease, respectively. A significant decrease in HTPs use was observed in adults with asthma.

After adjusting for confounders, the adjusted ORs and 95% CIs of HTPs use in current cigarettes smokers were 2.88 (1.86–4.47) for COPD, 1.23 (0.99–1.52) for asthma and 5.81 (3.12–10.82) for ACO compared to those without each disease, respectively. A significant increase in HTPs use was observed in adults with COPD and ACO.

## Discussion

In the present study, we evaluated the association between HTPs use and COPD, asthma, and ACO in adults aged 40 years or older among never, past, and current cigarettes smokers. The results of this study indicate significantly higher HTPs use in current cigarettes smokers with COPD and ACO compared to those without these respiratory diseases, although this difference is not significant in those with asthma.

In 2018, the National Health and Nutrition Survey reported that 22.1% of male and 14.8% of female current cigarette smokers were HTP users [27]. Our study indicated a higher HTPs use in current cigarettes smokers with COPD, asthma, and ACO, in particular, current cigarettes smokers with COPD and ACO had HTPs use of more than 50%, although its proportion was 0.8% and 15.6% as a whole in never and past cigarettes smokers, respectively. Previous reports showed that the dual use of HTPs and cigarette was 13.9%–18.4% in Korea [28, 29] and 10.5% in Japan [23], and demonstrated that HTPs use was significantly higher among current cigarette smokers compared to former and never cigarette smokers with or without respiratory diseases [30] and current cigarette smoking itself was the strongest concurrent predictor of HTPs use [31]. As the reason they are easily becoming dual users of cigarettes and HTPs, Lau et al. showed that daily cigarette users and exclusive HTP users were both heavily nicotine-dependent compared with exclusive, non-daily cigarette users [27]. HTPs might be used more often as a way for cigarette smokers and dual users to quit smoking [21, 27] but HTP users or dual users were less likely to quit tobacco compared to exclusive cigarette smokers (risk ratio, 0.77; 95% CI, 0.61–0.97) in a Japanese workplace [32]. Thus, dual users of cigarettes and HTPs may be less likely to attempt quitting both products and show lower motivation and self-efficacy to quit HTPs use [28, 31].

Our study showed higher rates of HTPs use and/or dual-use among adults with COPD, asthma, and ACO compared to those without these respiratory diseases. In particular, a significant increase in the adjusted ORs of HTPs use in adults with COPD and ACO and an increase in the tendency (adjusted OR, 1.23; 95% CI, 0.99–1.52, *P* = 0.057) in those with asthma was shown, despite being extremely important for quitting smoking in adults with these respiratory diseases. Similarly, participants aged 15–73 years in the JASTIS 2019 study showed that the adjusted OR of both cigarettes and HTPs use for COPD and asthma was 7.46 (3.76–14.80) and 1.69 (1.18–2.41), respectively [11]. Meanwhile, a Korean study targeting adolescents showed that the rate of HTPs use among asthmatic smokers was approximately 4.4 times higher (12.0%) than that in non-asthmatic smokers, and the dual use of HTPs and cigarettes was strongly associated with an increased risk of asthma (OR, 4.38; 95% CI, 2.44–7.87) [33, 34]. Thus, adults with these respiratory diseases tend to continue to smoke cigarettes, despite cigarette smoking being a major cause of COPD and ACO, which might be explained by a stronger nicotine dependence by long-term exposure to cigarette smoke in these adults, although this explanation might not be necessarily applicable to those with asthma.

A higher proportion of HTPs use in adults with COPD, asthma, and ACO may be an important clinical issue. Hirai et al. reported that the behavior of switching from cigarettes to HTPs is a common reason for not quitting smoking in current cigarette smokers with COPD [9]. Meanwhile, HTPs use was negatively associated with abstinence from cigarettes, and a complete replacement of cigarettes with HTPs was unlikely [35, 36], although there is a report that nearly 60% of patients with COPD using HTPs abstained completely from cigarette smoking [37]. Therefore, our results indicate that not just switching from using cigarettes to HTPs, but quitting both cigarette smoking and HTPs is important, although the association between each type of HTP and these respiratory diseases may be evaluated in further studies.

Our data showed that former cigarettes smokers with COPD, asthma, and ACO were less likely to use HTPs compared to those without these respiratory diseases. Despite the importance of quitting smoking, Kioi et al. showed the rate of having no intention to quit smoking among current smokers to be 40.0% and 44.7% in those with COPD and asthma, respectively [7], whereas Odani et al. reported no significant changes in HTPs use between current smokers with and without an intention to quit smoking [21]. In addition, HTPs use may strongly lead to the relapse of and/or reusing of cigarettes among long term smokers that have quit smoking smokers and never smokers [13]. Therefore, it is difficult to quit HTPs in current cigarette smokers, and adults with these respiratory diseases must understand that quitting cigarette smoking is important in order to avoid HTPs use and that quitting cigarette smoking alone is the effective way to not be HTPs and/or dual users in adults with COPD, asthma, and ACO.

This study included a relatively large sample size and nationwide sampling design and demonstrated the current status of HTPs use and the importance of quitting smoking among middle-aged and older adults with these respiratory diseases in Japan.

This study has several limitations. First, this study was a self-reported internet survey and the clinical diagnosis was self-reported as obtained from questionnaires. Previously, it was reported the self-reported diagnosis of asthma correlated well with the diagnosis of asthma [38], whereas, the self-reported diagnosis of COPD might have low sensitivity despite having high specificity [39]. Thus, the number might be over- or under-estimated. However, we selected adults over 40 years of age to confirm the diagnosis of COPD and ACO, and responders with discrepancies or inconsistencies in their answer were excluded to improve the accuracy of the study, therefore, we believe that the internet validity of the findings has been improved. In addition, the incidence of COPD in this study was only 1.3%, but the reported prevalence of COPD in 2017 was 0.2% according to the Japanese Ministry of Health, Labour and Welfare patient survey, although the prevalence of COPD in Japanese individuals over 40 years of age was estimated to be 8.6% [40]. Therefore, the extraction rate of COPD in the present study may be reasonable. However, we need to be very careful about the possibility of under- or over-estimation and the potential for a significant bias with regard to the specific diagnosis. Second, an internet survey is beneficial and can gather a large number of participants, but may involve a participant bias. In other words, the study participants might not be generalizable to the population with limited internet access or internet literacy and might not be fully representative of the general population. Thus, the distribution of the population might be imperfect and may involve a selection bias. Third, ACO was defined based on the presence of both COPD and asthma, but it may be unclear whether the definition of ACO used in this study accurately represents patients with ACO in actual clinical practice. Furthermore, we think that the concept of ACO is clinically meaningful due to significant differences in the treatment strategies [41], but we need to pay attention to the fact that there exists active discussion about the significance of ACO [42]. Finally, this research is based on cross-sectional data, which makes it difficult to consider causal inference; thus, further investigation by longitudinal studies should be considered.

In conclusion, adults with COPD, asthma, and ACO aged 40 years or older are more frequent HTP users among current cigarettes smokers and less HTP users among former cigarettes smokers compared to those without these respiratory diseases. Thus, we believe that complete cessation of both cigarettes smoking and HTPs use is the only way to achieve complete smoking cessation in adults with COPD, asthma, and ACO, and adults with these respiratory diseases need to make greater efforts to quit smoking.

## Data Availability

The datasets used for the current study are available from the corresponding author on reasonable request.

## Abbreviations

COPD: Chronic obstructive pulmonary disease
ACO: Asthma-COPD overlap
HTPs: Heated tobacco products
JASTIS: Japan Society and New Tobacco Internet survey
CI: Confidence interval
OR: Odds ratio
FEV_1.0_: Forced expiratory volume in one second
